# Interplay between Narrative and Bodily Self in Access to Consciousness: No Difference between Self- and Non-self Attributes

**DOI:** 10.3389/fpsyg.2017.00072

**Published:** 2017-01-31

**Authors:** Jean-Paul Noel, Olaf Blanke, Andrea Serino, Roy Salomon

**Affiliations:** ^1^Laboratory of Cognitive Neuroscience, Faculty of Life Science, Brain Mind Institute, Ecole Polytechnique Federale de LausanneLausanne, Switzerland; ^2^Center for Neuroprosthetics, Ecole Polytechnique Federale de LausanneLausanne, Switzerland; ^3^Vanderbilt Brain Institute, Vanderbilt University, NashvilleTN, USA; ^4^Department of Neurology, University HospitalGeneva, Switzerland; ^5^Department of Psychology, Alma Mater Studiorum – Università di BolognaBologna, Italy; ^6^Gonda Multidisciplinary Brain Research Center, Bar-Ilan UniversityRamat Gan, Israel

**Keywords:** self, criterion bias, peripersonal space, multisensory, audio, consciousness

## Abstract

The construct of the “self” is conceived as being fundamental in promoting survival. As such, extensive studies have documented preferential processing of self-relevant stimuli. For example, attributes that relate to the self are better encoded and retrieved, and are more readily consciously perceived. The preferential processing of self-relevant information, however, appears to be especially true for physical (e.g., faces), as opposed to psychological (e.g., traits), conceptions of the self. Here, we test whether semantic attributes that participants judge as self-relevant are further processed unconsciously than attributes that were not judged as self-relevant. In Experiment 1, a continuous flash suppression paradigm was employed with “self” and “non-self” attribute words being presented subliminally, and we asked participants to categorize unseen words as either self-related or not. In a second experiment, we attempted to boost putative preferential self-processing by relation to its physical conception, that is, one’s own body. To this aim, we repeated Experiment 1 while administrating acoustic stimuli either close or far from the body, i.e., within or outside peripersonal space. Results of both Experiment 1 and 2 demonstrate no difference in breaking suppression for self and non-self words. Additionally, we found that while participants were able to process the physical location of the unseen words (above or below fixation) they were not able to categorize these as self-relevant or not. Finally, results showed that sounds presented in the extra-personal space elicited a more stringent response criterion for “self” in the process of categorizing unseen visual stimuli. This shift in criterion as a consequence of sound location was restricted to the self, as no such effect was observed in the categorization of attributes occurring above or below fixation. Overall, our findings seem to indicate that subliminally presented stimuli are not semantically processed, at least inasmuch as to be categorized as self-relevant or not. However, we do demonstrate that the distance at which acoustic stimuli are presented may alter the balance between self- and non-self biases.

## Introduction

The inherent complexity of the construct of the self, has led to distinct conceptualizations typically including several levels of “selfhood” (James, 1890; Damasio, 1996, 1999; Parnas, 2003; Northoff and Bermpohl, 2004; Zahavi, 2005). At a foundational level of the self is a pre-semantic and non-conceptual understanding of the bodily self, which may serve as a scaffold for more complex and abstracts understandings of the self (Damasio, 1996, 1999; Gallagher, 2005; Legrand, 2006, 2007; Blanke and Metzinger, 2009). The pre-reflective level of self-representation has been suggested to be derived from multisensory-motor correspondences (Blanke, 2012; Blanke et al., 2015) and is thought to be developed at an early age (Rochat, 1995, 2003; Filippetti et al., 2013). It is only at later stages of development that higher-level semantic representations of the self – such as the narrative or autobiographical self (Northoff and Bermpohl, 2004; Northoff et al., 2006) – representing reflective conceptualizations of the self across time and space are established (Rochat, 1995, 2003; Damasio, 1996, 1999; Baumeister, 1998). Thus, a core self-representation is suggested to be grounded in the bodily self, while semantic and psychological representations of the self are developed later, putatively scaffolded upon the bodily-self representation, and involving memory and cognitive constructs of oneself across time and space (Arzy et al., 2008; Peer et al., 2015).

Both the bodily and the narrative conceptions of the self have been described as a fundamental construct bearing on human survival (Keenan et al., 2000; Humphrey, 2006) and correspondingly, the processing of self-relevant information has been shown to enjoy a privileged status (Yu and Blake, 1992; Tong and Nakayama, 1999; Sugiura et al., 2000; Salomon et al., 2011, 2013). At the sensory level, an example of this privileged self-processing can be observed in the so-called “cocktail party effect” (Cherry, 1953; Wood and Cowan, 1995) in which one’s name is automatically picked up by the auditory system among a sea of sensory noise (see Schäfer et al., 2016, for a recent demonstration of self-prioritization effects across various sensory modalities). A similar “self-reference effect” (Rogers et al., 1977) has been described for mnemonic processes in which information that is encoded as being self-relevant is a posteriori better retained (Sui and Humphreys, 2015). Further, it has recently been shown that even an arbitrary and externally imposed relationship between stimuli and the self causes enhanced processing of these stimuli (Sui et al., 2012, 2013; Stein et al., 2016). Thus, seemingly both at the sensory and at higher-order cognitive levels, self-related stimuli may enjoy of enhanced or prioritized access to awareness.

The bifurcation between the study of the bodily-self and a narrative-self has nonetheless arguably led to neglecting possible interactions between the sensory signals which are at the basis of the bodily self and higher-order level processes relating to the narrative self (see Canzoneri et al., 2016 for a recent exception).

Regarding the bodily-self, recent studies have shown that coupling masked visual stimuli with related bodily signals affects the emergence of the visual stimuli into awareness. Such effects have been shown for proprioceptive (Salomon et al., 2013), tactile (Lunghi et al., 2010; Lunghi and Alais, 2013; Salomon et al., 2015a), vestibular (Salomon et al., 2015b), and interoceptive signals (Salomon et al., 2016b). Our group, for example, has demonstrated that pairing unseen visual stimuli with a congruent body posture causes the unseen visual stimuli to break continuous flash suppression (CFS; Tsuchiya and Koch, 2005; Jiang et al., 2007; Stein et al., 2011) faster than when the stimuli is paired with incongruent proprioceptive signals (Salomon et al., 2013). That is, when participants are presented with highly visible and salient stimuli to their dominant eye (e.g., “Mondrians”), and visual stimuli with a lesser contrast or salience to their non-dominant eye (e.g., CFS paradigm), this latter stimulus will break the Mondrian suppression and thus access consciousness more readily when paired with a congruent (vs. incongruent) body posture. Further, the co-localization of sound and visual stimuli has equally been demonstrated to thrust this latter one into awareness under a breaking CFS paradigm (Aller et al., 2015). Similarly, using a related visual dichoptic presentation paradigm, namely, binocular rivalry (see Alais and Blake, 2005, for review), Lunghi et al. have demonstrated striking interactions between the administration of touch and/or audio-tactile stimulation and congruently presented visual stimuli in propelling the latter one into awareness (Lunghi and Alais, 2013; Lunghi et al., 2014). Further, sensory signals presented not only at the body, but also near the body, in the peripersonal space (PPS; Serino et al., 2015), have been demonstrated to heavily influence self-processing (Blanke et al., 2015; Noel et al., 2015a, 2016; Salomon et al., 2016a), and to reference conceptual processing (Canzoneri et al., 2016). Thus, sensory signals from the body or within the PPS may affect the processing of visual and self-related stimuli (see Faivre et al., 2015 for review).

On the other hand, in relation to the preferential processing of the semantic self, the scope and limits of unconscious semantic processing have revealed contradictory results – arguably in great measure as a consequence of the utilization of different experimental paradigms (Hesselmann and Moors, 2015), but also due to distinct levels of association between physical and psychological conceptions of the self. While researchers employing unconscious priming (which may arguably primarily index non-conscious processing, as opposed to access to consciousness, as CFS does; Stein et al., 2011) have demonstrated powerful effects of unconscious social primes influencing posterior behavior (Bargh et al., 1996; Dijksterhuis and Van Knippenberg, 2000), these subliminal effects are not always present – in particular when scrutinizing the possibility of observing subliminal semantic processing (Zimba and Blake, 1983; Blake, 1988; Kang et al., 2011). In fact, researchers have argued that the default stance should be not to expect much (self or non-self) semantic unconscious processing during incongruent dichoptic presentation (Hesselmann and Moors, 2015), and Blake (1988) inclusively reported that even the first name of a specific observer, which represents a fundamental constituent of the semantic self (Joubert, 1991) proved insufficient to alter dominance of one percept (self) over another (non-self) under a condition of dichoptic stimulation. Tacikowski and Ehrsson (2016) have recently reported a similar effect, in that even one’s own name, when masked, was insufficient to act as a self-prime.

Contrarily, recent studies utilizing a breaking CFS paradigm have demonstrated a host of effects presumably relying on unconscious semantic processing. Sklar et al. (2012), for instance, have shown that semantically incoherent expressions broke suppression (that is, “emerged” into visual awareness) more readily than semantically coherent expressions. Similarly, these researchers showed that increasing the negative affect of expressions lowered suppression time significantly, and that effortful arithmetic equations can be solved without awareness (Sklar et al., 2012). Yet other groups have demonstrated that emotional information is processed during suppression (Yang et al., 2007), scene congruency information can be extracted in the absence of visual awareness (Mudrik et al., 2011), and that word meaning (Costello et al., 2009) and word valence (Yang and Yeh, 2011) can be processed unconsciously. Further, particularly with regard access to consciousness of self-relevant information under the context of breaking CFS, Geng et al. (2012) have demonstrated that one’s own face is detected more rapidly than other faces, while Stein et al. (2016), did not show the analogous effect when a visual stimulus (gabor) was arbitrarily labeled “You” as opposed to “Other” (Stein et al., 2016).

Overall, the recent demonstrations of semantic processing under CFS beget the question whether an unconscious semantic representation of the self can be processed unconsciously. The prospect of revealing semantic self-processing unconsciously has been troublesome, but evidence indicates that this prospect may be bolstered by investigating semantic self-processing in combination with lower-level understandings of the bodily self. Here we investigated if participants are (i) able to process self-relevant semantic stimuli unconsciously and (ii) if this is modulated by task irrelevant stimuli, which tap into a specific multisensory dimension of the bodily self, i.e., the PPS. In a first experiment, we present participants with self-attributed personality trait to their non-dominant eye, while presenting dynamic high-contrast stimuli to their dominant one in order to mask the self-attribute. Participants were then to categorize the presented but unseen words as either self-related or not. We tested whether participants were able to semantically process self-related information inasmuch to appropriately categorize the words as self-relevant or not. Successful categorization of unseen words as either self-related or not is taken to index unconscious self-processing and is thus the dependent variable of interest here. That is, we questioned whether subjectively invisible words could be nonetheless accurately classified as self vs. non-self. In a second experiment, we tested whether such putative self-effect could be modulated by a low-level manipulation prioritizing the representation of the bodily self. One key aspect of bodily self is its link to the body and to the space immediately surrounding it, i.e., the PPS (Blanke, 2012; Blanke et al., 2015; Noel et al., 2015a, 2016; Canzoneri et al., 2016; Salomon et al., 2016a). Thus, here, we tested the hypothesis that presenting an acoustic stimuli within, as opposed to outside, PPS may bolster unconscious semantic self-processing. In this manner, we aimed at testing a direct link between bodily and narrative representations of the self and how they interact to modulate awareness.

Continuous flash suppression was utilized in order to mask presented stimuli as it has been suggested that this paradigm allows for some sort of non-conscious physical/perceptual (Geng et al., 2012), but not the psychological/conceptual self-processing (Stein et al., 2016). Hence, it represents an ideal candidate to reveal prioritized non-conscious processing by the psychological self, when this latter one is related to its physical conception. Lastly, self-rated personality attributes were used as these tap into the core of the psychological self while equally being malleable in time and self-selected by the participants themselves. That is, in contrast to other experiments that have used a fixed set of stimuli (e.g., Blake, 1988), here we aimed at having a set of words that were always the same yet for some participants fell within the “self” category, while for other fell in the “non-self” category.

## Materials and Methods

### Participants

Forty-five native French speakers (nine females, mean age = 22.2 years old, range = 18–30 years old) completed a self-attribution questionnaire (see below), out of which 23 (six females, mean age = 21.4 years old, range = 18–27 years old) partook in both Experiment 1 and Experiment 2. The rest of participants were discarded from participation in the experimental phase, as their self-reports (see below) showed a bias in the attributes they categorized as “self” or “non-self” in terms of either the word-length, or the frequency of the select word in the French language. All participants were right-handed, had normal or corrected-to-normal visual acuity, and reported normal hearing. The study was approved by the Brain Mind Institute Ethics Committee for Human Behavioral Research of the EPFL, and conducted in line with the Declaration of Helsinki. All participants gave informed consent prior to participation and were remunerated with 20 Swiss Francs for their time.

### Self-attribution Reports

#### Materials, Procedure, and Analyses

Anderson’s Likableness ratings of 555 personality-trait words (Anderson, 1968) were sorted from most to least likable (according to the mean values in Anderson, 1968), and the middle 200 words (word 178: *hopeful* to word 378: *frustrated*^1^) were translated into French by three native French speakers. If translators did not independently agree on the translation of an attribute, they were asked to reach a consensus. This middle section of Anderson’s personality-trait words was selected in order to increase between-subject variability in self-attribution reports (see below) while also minimizing the within-word likability variance.

Forty-five participants completed, online and at their own time of convenience, a self-attribution questionnaire, which was constituted of the translated 200 personality-trait words. Participants were asked to rate on a Likert Scale (1 = completely not-self to 7 = completely self) whether the attributes described themselves (self) or not (non-self), and whether they liked the word (also on a Likert Scale). No further instruction was given to participants completing the questionnaire in terms of what they should consider when judging whether a particular attribute described them or not. Word order was randomly shuffled between participants. Upon receiving participant’s response to the self-attribution questionnaire, for each subject individually, the list of word was sorted from most to least “self,” and the top and lower 50 words were compared by means of a paired *t*-test (alpha set at 0.05, two-tailed) for word-length, frequency in the French language (word frequency was obtained from http://www.lexique.org/listes/liste_mots.php), and likability. If for a particular subject the group of self and non-self words differed any of the variables stated above (length, frequency, or likability), the participant was not invited to partake in Experiments 1 and 2.

### Experiment 1

#### Materials and Apparatus

**FIGURE 1:**
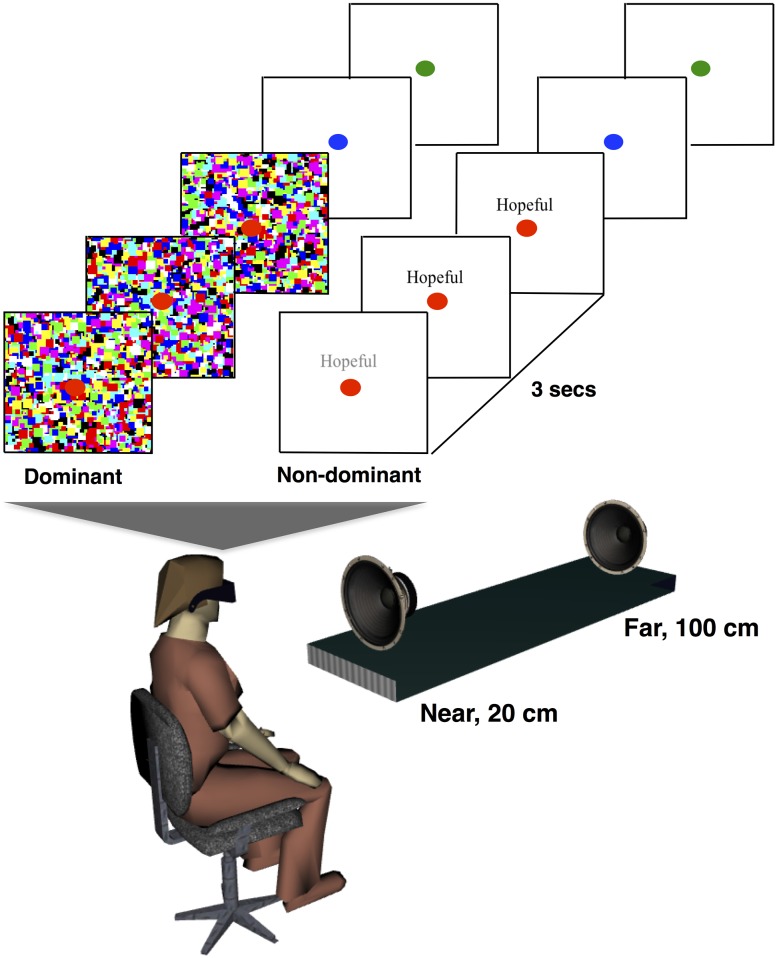
**Experimental setup and design.** In Experiment 1, participants were presented with either self or non-self attributes to their non-dominant eye (depicted; “Hopeful”), while high-contrast mondrians were flashed to their dominant eye in order to make the attributes imperceptible. Attributes were presented for 3 s while participants fixated on a red dot. Subsequently, the dot turned blue, which indicated that participants were to respond to the question, “Was the additional stimuli self-related or not?” and then green, which indicated that subjects were to answer to the question, “Was the additional stimuli presented above or below fixation?”. In Experiment 2, the same protocol as in Experiment 1 was repeated, but additionally a white noise sound was presented for a second (the last second of the attribute presentation) either from the near or from the far speaker.

Visual stimuli consisted of high-contrast dynamic noise patches suppressors (Mondrians; Jiang and He, 2006; Hesselmann and Malach, 2011) and target stimuli. The target stimuli consisted of one of the personality-trait words (either “self” or “non-self”) in black Times New Roman, font 12 (example in **Figure 1**; “Hopeful”). Background was white. Mondrians (noise stimuli) were flashed at 10 Hz to the participants’ dominant eye, and the targets were presented simultaneously to the other eye. A red fixation point was presented to both eyes. The target words we presented either above or below the fixation point in a randomized fashion. Stimuli were presented using ExpyVR^2^, an in-house custom-built multimedia stimuli presentation software developed with Python 2.6 and the Open Graphics Library v.2.2. The stimuli were viewed via a Head-Mounted Display (HMD: VR1280, Immersion Inc, SXGA, 60° diagonal field of view, refresh rate 60 Hz). Participants’ responses were gathered via button-press on a gamepad (XBOX 360 controller, Microsoft, Redmond, WA, 215 Hz sampling rate).

#### Procedure

Ten to 15 days after completion of the self-attribution report, subjects were brought into the lab for completion of the experimental phase (Experiments 1 and 2, which were ran in a counter-balanced order across participants). Participants were first tested for ocular dominance via the Miles test (Miles, 1930). Then, they were instructed that on each trial, in addition to the fixation point, which they were to gaze at, they would see a random pattern of colorful squares. They were equally told that, during some trials they would additionally see “a sequence of letters.” They were instructed to press, during the trial, a button on a gamepad if they saw this additional stimulus. Those trials were indexed as having broke suppression, and removed from analyses for the stimuli categorization and location questions (see below), as we are interested in trials for which no subjective experience of the words was reported (i.e., unconscious self-processing). Then, as portrayed in **Figure 1** (upper), subjects were informed that upon completion of a trial, they would see the fixation point changing color: first blue, which indicated participants were to answer to the question “Was the additional stimuli self-related or not?”, then green, which indicated that subjects were to answer to the question “Was the additional stimuli presented above or below fixation?” The questions were always asked in this order, and participants were reassured that if they had not seen anything (apart from the Mondrians and the fixation point) they were to simply guess. The question related to stimuli location (above or below fixation) was employed as a probe that some minimal form of processing was present when suppressing self-attributes. Participants responded via button-press, which mapping (self/non-self and above/below) was randomized across participants.

Trial presentation (Mondrians and personality-trait words) lasted for 3 s. Contrast of the target stimuli was ramped linearly from zero to full contrast over the 1 s. Participants were given unlimited time to answer both questions (Self-Attribution and Stimulus Location). Inter-trial interval was randomly shuffled between 1 and 1.5 s. The experiment consisted of 200 trials (presentation of each of the 100 participant-specific personality-trait attributes – 50 self and 50 non-self – twice. Once above and once below fixation). Total experimental time was about 30 min.

#### Analyses

Participants’ responses were first analyzed in terms of percentage of trials in which subjects broke suppression for self vs. non-self words, as well as for words presented above vs. below fixation. Then, subsequently to discarding the trials in which participants broke suppression (e.g., participants were conscious of the stimuli), Signal Detection Theory (SDT) analysis (sensitivity – d′, and criterion – *c*) was applied to both the Self-Attribution and Stimulus Location questions. Null effects were assessed using JZS Bayes factor (BF) tests with default prior scales (Rouder et al., 2012) so that a BF < 0.33 implies substantial evidence for the null hypothesis, 0.33 < *B* < 3 suggests insensitivity of the data, and *B* > 3 implies substantial evidence for the alternative hypothesis (see Dienes, 2011).

### Experiment 2

The material and apparatus, as well as the procedure and analyses used in and applied to Experiment 2 (which, again, was counter-balance in order with Experiment 1 across participants) followed largely those of Experiment 1, for the exception of the following.

In order to probe at whether an implicit association with the bodily self, by delivery of auditory stimuli within the PPS (Noel et al., 2015a; Serino et al., 2015; Salomon et al., 2016a), would boost non-conscious self-processing, acoustic stimuli (white noise) were presented both close to, and far from, the body. Specifically, as depicted in **Figure 1** (lower), a speaker (Near) was placed 20 cm in front of the participants in the anterior-posterior axis, at his or her midline and at sternum level, while a second speaker (Far) was placed 100 cm away (at the same horizontal and elevation level). The speakers emitted white noise for the last second of trial presentation. They were both regulated in order to produce 70 dB(A) of sound measured at the participants’ ear. White noise was utilized (as opposed to more naturalistic stimuli) as this stimuli has been demonstrated to most effectively drive neurons encoding for PPS (Fogassi et al., 1996), and the onset of the acoustic stimuli was offset with respect of onset of visual stimuli in order to minimize the effect audio onset can have in thrusting a visual stimuli into awareness (Aller et al., 2015). Experiment 2, as Experiment 1, also consisted of 200 trials (presentation of each of the 100 participant-specific personality-trait attributes twice. Once while a near sound was presented, and once while a far sound was presented (randomized on a trial by trial fashion). Total experimental time was about 30 min.

## Results

### Experiment 1

#### Breaking Suppression

A repeated measures ANOVA was ran on the percentage of occasions in which self vs. non-self words broke suppression, as well as for words presented above vs. below fixation. Results revealed no significant difference between levels for neither Self-Attribution [*F*(1,21) = 0.241, *p* = 0.83] or Stimulus Location [*F*(1,21) = 0.832, *p* = 0.64], nor an interaction between these variables [*F*(1,21) = 0.828, *p* = 0.40]. Bayesian statistics demonstrated a BF of 0.23 in the case of the former and 1.15 in the case of the latter. Thus, in the case of the frequency with which self vs. non-self words broke suppression, data implies substantial evidence for the null hypothesis. Overall, stimuli broke suppression in about a fifth of the trials (self: *M* = 20.0%, *SE* = 6.4%; non-self: *M* = 19.7%, *SE* = 6.4%; above: *M* = 19.0%, *SE* = 6.5%; below: *M* = 20.7%, *SE* = 6.3%).

#### Self-Attribution

Sensitivity and criterion analysis on participants’ response to the question; “Was the additional stimuli self-related or not?” was effectuated by means of purpose-made scripts in MATLAB (equations follow Macmillan and Creelman, 2005) and subsequent one-sample *t*-test comparison to zero (meaning no sensitivity and no bias, respectively for *d’* and *c*). The “Self” category was defined as “target,” while the “Non-Self” category was used as “noise.” Results revealed that participants were not able to discriminate between self and non-self words [*d’*; *M* = -0.49, *SEM* = 0.27; *t*(22) = -1.35, *p* = 0.32], nor did they show any bias [*c*; *M* = 0.03, *SEM* = 0.06; *t*(22) = -0.60, *p* = 0.55]. In the case of *d’*, Bayesian statistical analysis suggests a lack of sensitivity data (BF = 0.82), nonetheless, in the case of the response bias Bayesian statistics provided substantial evidence for the null hypothesis (BF = 0.25).

#### Stimulus Location

In terms of subjects’ responses to the question about Stimulus Location; “Was the additional stimuli presented above or below fixation?”, and defining “Above” as “target” and “Below” as “noise,” findings revealed that participants were indeed able to discriminate between the elevation of the personality traits presented [*d’*; *M* = 1.24, *SEM* = 0.19, *t*(22) = 5.82, *p* < 0.001] and did not show a criterion bias [*c*; *M* = 0.10, *SEM* = 0.05; *t*(22) = -1.24, *p* = 0.12]. In the case of the latter, the Bayes factor (BF = 1.1) seems to indicate a lack of sensitivity in the data.

### Experiment 2

#### Breaking Suppression

Repeated-measures ANOVAs were conducted on the percentage of occasions in which self vs. non-self words broke suppression, as well as for word location (Above vs. Below fixation), as a function of the location of sound presentation (Near or Far). Concerning the frequency in which self vs. non-Self words broke suppression as a function of the distance of sound presentation, results revealed no main effect, neither for Self-Attribution [*F*(1,21) = 0.83, *p* = 0.37], or Sound Location [*F*(1,21) = 0.04, *p* = 0.92], nor an interaction between these variables [*F*(1,21) = 0.99, *p* = 0.33]. In the case of the main effect Bayesian statistics demonstrated insensitive data (all BFs > 0.62), while in the case of the interaction these statistics provide substantial evidence for the null hypothesis (BF = 0.18).

A similar pattern of results was observed for the frequency with which words presented Above vs. Bellow fixation broke suppression, as a function of Sound Location. The repeated-measures ANOVA showed no main effect of Word Location [*F*(1,21) = 0.94, *p* = 0.35] or Sound Location [*F*(1,21) = 0.745, *p* = 0.41], nor an interaction between these variables [*F*(1,21) = 0.56, *p* = 0.57]. Bayesian Factors demonstrated substantial evidence for the null effect in the case of breaking suppression for attributes presented above vs. below fixation (BF = 0.22), while in the other cases Bayesian factors demonstrated the insensitivity of the data (all BF > 0.38).

#### Self-Attribution

**FIGURE 2:**
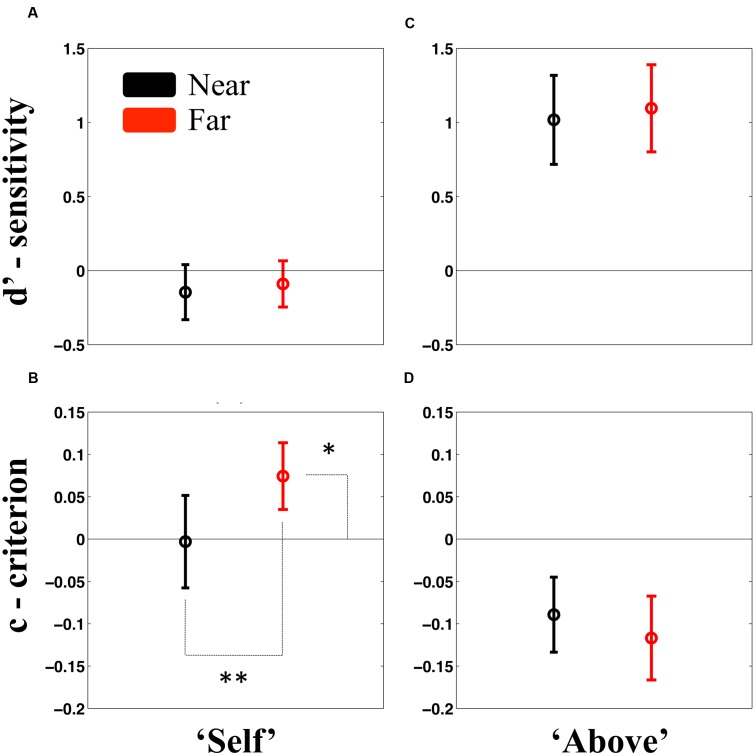
**Results from Experiment 2.** Results revealed no difference in sensitivity to either categorizing attributes as “Self” or “Non-Self” **(A)**, or for categorizing these as being “Above” or “Below” fixation **(C)**, regardless whether acoustic stimuli was presented near (black) or far (red) from the participant. Importantly, participants did exhibit a significantly different from zero sensitivity in categorizing the location at which stimuli were presented. In terms of criterion bias, however, results did reveal that participants adopted a more conservative approach to categorizing an unseen attribute as “Self” when sounds were presented far (red) as opposed to near (black) **(B)**. Lastly, participants showed no difference in response bias in terms of stimuli elevation when sounds were presented wither near or far from them **(D)**. Error bars represent ±1 *SEM*. ^∗^*p* < 0.05; ^∗∗^*p* < 0.01.

As for Experiment 1, the analysis of the self-attribution question was performed by SDT. We compare sensitivity and criterion bias across Sound Locations by means of a paired-samples *t*-test, and subsequently, if Sound Location significantly modified either the sensitivity or criterion to Self or Non-self attributed words, we compared these parameters to the zero-baseline. As portrayed in **Figure 2A**, results revealed that regardless of the Sound Location, participants were not able to discriminate between Self and Non-Self words [*t*(21) = -0.58, *p* = 0.56; *d′*_near_, *M* = -0.14, *SEM* = 0.19; *d′*_far_, *M* = -0.08, *SEM* = 0.16], as in Experiment 1. Further, Bayesian statistics demonstrated substantial evidence for the null hypothesis, both for the case of close sounds (BF = 0.29) and far sounds (BF = 0.26).

In terms of the criterion, however, as shown in **Figure 2B**, findings did demonstrate a significant difference between Sound Location levels [*t*(21) = 3.76, *p* < 0.01], revealing a more conservative approach for identifying “Self” when a sound was presented Far (*M* = 0.07, *SEM* = 0.02), as opposed to when the sound was presented Near (*M* = -0.003, *SEM* = 0.05) to the participant. Comparisons to zero, indicated that the “far criterion,” was indeed significantly different from no bias [*t*(21) = 1.80, *p* = 0.03], while the “near criterion” was not [*t*(21) = -0.05, *p* = 0.95]. These findings are further supported by the demonstration that in the case of the latter (near sounds), but not the former (far sounds), Bayesian statistics provided substantial evidence in favor of the null hypothesis (BF = 0.22).

#### Stimulus Location

With regard whether the target stimuli was presented above or below fixation, analysis demonstrated no significant difference between Sound Location levels [*t*(21) = -0.31, *p* = 0.75, BF = 0.32]. It must be noted, however, that as depicted in **Figure 2C**, and replicating the results from Experiment 1, participants did prove to be overall sensitive to the location of the target stimuli [*d’*; *M* = 1.05, *SEM* = 0.29, *t*(21) = 4.622, *p* < 0.001, two-tailed one-sample *t*-test vs. zero].

Lastly, contrarily to the case of the Self-Attribution reports, and as shown in **Figure 2D**, participants did not show a systematic bias in their response to the elevation of target stimuli as a consequence of Sound Location [*t*(21) = 0.338, *p* = 0.73]. BF was equal to 0.32, providing evidence for the postulation that participants were equally likely to categorize unseen visual stimuli as happening above or below fixation regardless of whether an acoustic stimulus was presented near or far from them.

## Discussion

In the present study, we first posed the question whether self-relevant (vs. non-self relevant) semantic information is preferentially unconsciously processed. In addition, we tested whether such self-effect could be modulated by priming a bodily-self representation via the presentation of a near sound within PPS.

In line with previous studies investigating semantic processing outside awareness (Zimba and Blake, 1983; Blake and Logothetis, 2002; Dehaene et al., 2006), our results indicate that masked words were not processed at the semantic level – at least inasmuch as to reveal a self-processing advantage (see Tacikowski and Ehrsson, 2016, as well as Stein, et al., 2016). This was true both in the case when no sounds were presented, and when sounds were presented either close to or far from the participant. It is important to highlight, however, that participants were able to correctly localize the elevation of the presented unseen visual stimuli, and thus, the lack of evidence for semantic processing is apparent even in light of clear evidence for lower-level subliminal processing. Blake (1988) also reported that even the first name of a specific observer, which represents a fundamental constituent of the semantic self (Joubert, 1991), proved insufficient to alter dominance of one percept (self) over another (non-self) under a condition of dichoptic stimulation. In contrast to the traditional view, however, an array of recent studies employing the b-CFS paradigm seem to demonstrate high-level processing of stimuli suppressed from conscious experience. Most notably, Jiang et al. (2007) showed that words that were visually familiar to a specific population’s lexicon broke suppression more readily than foreign words. Costello et al. (2009), similarly demonstrated that semantic priming subsequently accelerated breaking suppression of related words, and finally Yang and Yeh (2011) reported that the affective connotation of an unseen word altered the duration for which they could be maintained under suppression. It is under this context – one that has previously indicated the presence of semantic processing outside of awareness (Jiang et al., 2007; Costello et al., 2009) and even evidencing congruency priming under complete unawareness (Faivre et al., 2014; but see Noel et al., 2015b), that the current findings are important. It may be that the familiarity of words and their affective connotation can be processed and even integrated outside awareness, yet self-related and non-self related words are not differentially prioritized, at least inasmuch as this processing would entail a sensitive categorization of self and non-self words into their appropriate categories unconsciously. That is, although generally studies employing dichoptic presentation methods other than CFS have reported limited unconscious semantic processing (e.g., Blake, 1988), the recent surge in popularity of the CFS method and the numerous findings indicating unconscious processing under this paradigm has renewed interest in unveiling unconscious semantic processing. In the current study, we thus utilize the CFS method in order to mask self-attributes, and our results demonstrate no prioritized unconscious processing of the self vs. the non-self words.

On the other hand, our results revealed an interesting result relating to a shift in the criterion bias as a consequence of the location of sound presentation. That is, while in Experiment 1 no criterion bias was present, findings from Experiment 2 revealed a more conservative self-categorization approach (or a more liberal other-categorization) when sounds were presented outside, as opposed to inside, their PPS. That is, individuals required more evidence in order to judge that the unseen word presented was “self” when a sound was presented far as opposed to near. This differential response bias as a consequence of sound location was specific for the self dimension, as we did not observe a differential response bias among sound locations for the indication of whether subliminal visual stimuli were presented above or below fixation. That is, arguably, the self-other distinction process was modified when an auditory stimulus was provided far from the self. This effect is reminiscent of behavioral (Tajadura-Jiménez et al., 2013; Teneggi et al., 2013; Noel et al., 2015a; Salomon et al., 2016a) and neuroimaging (Brozzoli et al., 2012, 2013) multisensory studies demonstrating a self-other differentiation process grounded on the representation of PPS. PPS, indeed, might represent a multisensory-motor representation of the self in interaction with the environment (Noel et al., 2014; Galli et al., 2015; Serino et al., 2015), and the PPS boundaries might define a first level of self-other distinction (Noel et al., 2016). In fact, Blanke et al. (2015) hypothesized that changes in the size of PPS neurons receptive fields might underpin alterations in states of bodily self-consciousness, and we have recently demonstrated that subjective changes in self-location co-vary with changes (i.e., spatial translations) in the representation of PPS (Noel et al., 2015a) and even for unconscious manipulations of bodily self consciousness (Salomon et al., 2016a). Here, we show that priming the PPS boundary by presenting an auditory stimulus within the PPS, as contrasted to a stimulus outside the PPS, can modify the criterion with which individuals categorize stimuli as self-related or other-related. That is, the administration of spatially restricted exteroceptive stimuli either within or outside the PPS alters cognitive processes with regard the self. We find this observation particularly interesting as it seems to imply a continuous and dynamic re-assessment to the self as external objects and events come in contact with our sensory modules. Importantly, this change in criterion as a consequence of sound location, however, did not modulate the emergence of self-attributes into awareness, but changed the post-perceptual criterion participants used to determine whether the invisible word was self-related or not.

## Conclusion

It appears that though bodily representations of the self are able to modulate access to visual awareness (Lunghi et al., 2010; Lunghi and Alais, 2013; Salomon et al., 2015b), they do not enhance non-conscious processing of the narrative self (or at least association with the PPS does not). Importantly, self-attributes did not emerge into awareness in a prioritized way, even when presented in synchrony with exteroceptive signals that did alter self-bias. This lack of effect could putatively emanate from an inability of unconscious semantic processing, and not from a lack of enhanced self-processing. Nonetheless this option is in contrast to the results of recent studies demonstrating semantic processing of subliminal stimuli masked by means of CFS (Yang and Yeh, 2011; Prioli and Kahan, 2015). In conclusion, our results demonstrate no evidence for differential processing of masked semantic self-relevant stimuli, but do demonstrate a shift in criterion bias as a function of whether stimuli are presented in the peri- or extra-personal space.

## Ethics Statement

The study was approved by the Brain Mind Institute Ethics Committee for Human Behavioral Research of the EPFL, and conducted in line with the Declaration of Helsinki. All participants gave informed consent prior to participation and were remunerated with 20 Swiss Francs for their time.

## Author Contributions

OB, AS, and RS conceived of the study, which was performed by J-PN. J-PN analyzed the data and wrote the manuscript, which was then edited by AS and RS. All authors approved the final version of the manuscript.

## Conflict of Interest Statement

The authors declare that the research was conducted in the absence of any commercial or financial relationships that could be construed as a potential conflict of interest.

## Footnotes

1 http://psy2.ucsd.edu/pwinkiel/words.txt

2 http://lnco.epfl.ch/expyvr
